# Miniaturized Shear Testing: In-Plane and Through-Thickness Characterization of Plywood

**DOI:** 10.3390/ma17225621

**Published:** 2024-11-18

**Authors:** Víctor Tuninetti, Moisés Sandoval, Juan Pablo Cárdenas-Ramírez, Angelo Oñate, Alejandra Miranda, Paula Soto-Zúñiga, Michael Arnett, Jorge Leiva, Rodrigo Cancino

**Affiliations:** 1 Department of Mechanical Engineering, Universidad de La Frontera, Temuco 4811230, Chile; jorge.leiva@ufrontera.cl; 2 Master Program in Engineering Sciences, Faculty of Engineering, Universidad de La Frontera, Temuco 4811230, Chile; m.sandoval27@ufromail.cl; 3 R&D Innovation Department, Eagon Lautaro S.A., Ruta 5 Sur Km 644, Lautaro 4860000, Chile; rcancino@eagon.cl; 4 Facultad de Arquitectura, Construcción y Medio Ambiente, Universidad Autónoma de Chile, Temuco 4810101, Chile; juanpablo.cardenas@uautonoma.cl (J.P.C.-R.); michael.arnett@uautonoma.cl (M.A.); 5 Ingeniería, Innovación y Desarrollo Sustentable Ltda., Cacique Cheuquellan 01689B, Temuco 4780000, Chile; 6 Department of Materials Engineering (DIMAT), Faculty of Engineering, Universidad de Concepción, Edmundo Larenas 315, Concepción 4070138, Chile; aonates@udec.cl; 7 College of Engineering, Architecture, and Design, Universidad San Sebastián, Campus Las Tres Pascualas, Lientur 1457, Concepción 4060000, Chile; alejandra.miranda.ve@gmail.com; 8 Program of Civil Engineering, Universidad de La Frontera, Temuco 4811230, Chile; p.soto10@ufromail.cl

**Keywords:** plywood, orthotropic, shear strength, mechanical characterization, miniaturized testing, *Pinus radiata*

## Abstract

This study addresses the challenges associated with conventional plywood shear testing by introducing a novel miniaturized shear test method. This approach utilizes a controlled router toolpath for precise sample fabrication, enabling efficient material use and data acquisition. Miniaturized samples, designed with double shear zones, were tested for τ_xy_, τ_xz_, and τ_yz_ configurations using a universal testing machine. Results revealed a mean ultimate shear strength ranging from 5.6 MPa to 7.3 MPa and a mean shear modulus ranging from 0.039 GPa to 0.095 GPa, confirming the orthotropic nature of plywood. The resulting shear behavior was determined with stress–strain curves correlated with failure patterns. The miniaturized tests effectively captured the material’s heterogeneous behavior, particularly at smaller scales, and demonstrated consistent load-bearing capacity even after substantial stress reduction, suggesting suitability for bracing applications. This method allows for increased sample sizes, facilitating robust data collection for developing and validating finite element models. Future work will focus on evaluating the scalability of the observed orthotropic behavior and data scatter at larger scales and assessing the potential for this method to replace conventional full-scale plywood shear testing.

## 1. Introduction

Plywood, a widely used engineered wood product, exhibits inherent orthotropic behavior due to the microstructure of the wood and the alternating grain direction of the layers that compose it. This material is renowned for its versatility in various applications, including construction and architecture, the furniture industry, transportation such as canoe manufacturing, and even historical aircraft fuselage in the early 1900s. Given its frequent use in construction, where it often replaces solid wood or structural envelope elements [1], determining the plywood response to loadings is practical for engineering design [2], particularly because wood is a composite material with anisotropic properties [3,4,5]. Several studies have highlighted the complexity of the behavior of wood panels under various environmental and loading conditions. Additionally, thermal treatment has been shown to enhance certain physical properties of plywood [5].

According to industry estimates, Chile is ranked among the top ten global producers of plywood [6]. Furthermore, Chile is the world’s largest producer of plywood made from radiata pine [7,8]. Within South America, the region’s largest radiata pine plantations, a key raw material for plywood manufacturing, are located in Chile [9,10,11], followed by Argentina, Uruguay, and Brazil. Chile’s prominent global position in the plywood industry [12] can greatly benefit from advances in the comprehensive characterization of its mechanical properties. These advances can optimize production processes and increase the industry’s competitiveness in international markets. In particular, a more detailed understanding of the orthotropic shear response of plywood can benefit the industry in several ways. Improved product quality control can be achieved by gaining a better understanding of the shear properties, allowing manufacturers to monitor and control critical production parameters more closely. This ensures consistent and predictable product performance for more accurate modeling and structural analysis, allowing engineers to optimize the design of plywood-based components and structures, leading to more efficient use of materials and improved structural integrity.

The production of structural plywood is a complex process that involves multiple factors, such as the wood species [5,13], veneer peeling method [14], temperature [3,13], moisture content [15], veneer roughness and surface quality [16,17,18], as well as the number and thickness of layers [3,19], type of adhesive [20], curing time [21], application speed, pressing time [22], and drying time. These panels are designed to provide high mechanical properties in all three structural directions, leveraging the orthotropic behavior of the material. The theoretical analysis of the elastic properties of plywood assumes predictable anisotropic behavior under idealized conditions [2]; however, demanding structural applications show dissimilar, non-homogeneity, and complex behavior predictions [23,24]. In addition, recent studies have also endeavored to increase the understanding of the deformation behavior of plywood panels with digital image correlation (DIC) and computed tomography (CT) to accurately determine the local deformations and deformation behavior of plywood panels [25].

There are various regulations governing the production of structural plywood. Among them are the U.S. standard PS1 for structural plywood [26] and the European standard EN 789, which addresses timber structures—test methods—determination of the mechanical properties of wood-based panels; EN 310, which concerns wood-based panels—determination of the modulus of elasticity in bending and bending strengths; and EN 314-1, which covers plywood—bonding quality—Part 1: test methods, which stand out. To comply with these regulations, the minimum thickness for structural plywood products available on the market is approximately 9 mm.

Characterizing the shear properties of plywood is critical for understanding its structural performance, enabling advancements in complex calculations and modeling processes, and facilitating virtual testing. The shear modulus and strength are essential parameters describing the linear elastic behavior of wood and engineered wood materials [27]. However, virtual tests require an appropriate characterization process to ensure reliable results. Traditional methods for evaluating the shear properties of plywood often involve full-scale tests [28,29,30], which can be costly and complex. These tests require specialized equipment, large samples, and considerable laboratory space. Additionally, producing a pure shear stress state in wood is extremely challenging due to the high degrees of anisotropy and heterogeneity present in the material [27,31,32,33].

The shear modulus and shear strength are essential for describing the linear elastic behavior of wood and wood-engineered materials, enabling advancements in complex calculations and modeling processes and facilitating virtual testing.

There are several standards that provide guidelines for shear testing in wood products, with the most notable being ISO 12465 [34], ASTM D1037 [35], ASTM D5456 [36], and EN 789 [37]. ASTM D1037 [35] and EN 789 [37] are widely used to determine the shear properties of wood parallel to the grain, whereas ASTM D5456 [36] focuses on the longitudinal shear properties of structural adhesives used in wood and other materials. On the other hand, ISO 12465 [34] describes the method for determining the shear strength of wood and its derivatives. These standards provide detailed procedures and data analysis methods for conducting the tests. However, key aspects regarding the production of miniaturized samples for shear evaluation have not been addressed.

Characterizing wood and engineered wood materials in shear with miniaturized sample size is challenging. Some authors have presented variations of miniaturized samples for shear testing but are limited to special fixtures, shear directions, and inherent in-homogeneities [38,39].

The geometries of the samples vary depending on the shear properties to be evaluated and the selected standard. Some of the most common geometries include specimens used to measure shear strength parallel to the grain and modified Iosipescu samples to evaluate shear strength in other directions. The lap shear test is employed to assess the bonding strength of plywood; however, this method only examines the bond adjacent to the tested veneer, ignoring other bonds that may be weaker and lead to structural failure [40]. Reported values of Shear strength for *pinus*-based plywood are 2.8 MPa and 2.2 MPa for 0° and 90° [41]; 0.97 MPa and 0.82 MPa for planar shear strength parallel to the grain and perpendicular, respectively [42]; and glue line shear strength of 2.41 of Phenol–formaldehyde bonded plywood [43]. A lab-scale plywood showing 1.3 MPa of shear strength under EN 314 leads to lower shear strength than an industrial board with 2.7 MPa [44].

On the basis of a rigorous literature review, the authors conclude that current standards and publications do not specify procedures for the fabrication of miniaturized samples that would allow for the characterization of shear properties in all three orthogonal planes, as required for constitutive models of highly anisotropic plywood panels. Current standards, which prioritize plywood bracing function, do not completely characterize its orthotropic behavior [45]. Standardized tests are limited to directions parallel (x-direction) and perpendicular (y-direction) to the outer grain and use large samples with complex setups, making large-scale implementation difficult. Furthermore, tests conducted on universal axial machines often require additional clamping devices or fixtures, increasing the complexity of the procedure, as noted by Ostapska et al. [39].

Furthermore, although methods such as the Iosipescu shear test have been adapted for wood-derived materials, they still focus on two principal directions and often require extensive sample preparation and advanced equipment, making scaling up the tests for comprehensive structural analysis difficult. The lack of standardized miniaturized testing protocols further complicates the characterization of shear in thinner panels or smaller samples, which are crucial for advanced modeling and virtual testing. These limitations in current standards highlight the need for more tailored procedures that can reliably characterize the anisotropic shear properties of plywood, especially for advanced structural applications. Although studies have been conducted on adhesive behavior in veneer bonding, research on the overall mechanical response of plywood, which is essential for structural design, remains limited. This lack of data on orthotropic properties complicates a realistic analysis of panel behavior in modular construction systems such as WikiHouse Skylark.

The proposed research aims to develop a novel miniaturized shear test sample and protocol to comprehensively characterize the orthotropic shear properties of radiata pine-based plywood panels in the three principal material directions. This work will address the limitations of current standards. The new miniaturized samples are assessed for efficient and reliable elastic and damage behavior determination. This is crucial for advancing complex modeling and virtual testing capabilities and optimizing the design of structural applications of plywood in innovative construction systems.

## 2. Materials and Methods

### 2.1. Design Process of the Miniaturized Shear Test Samples

**Figure 1:**
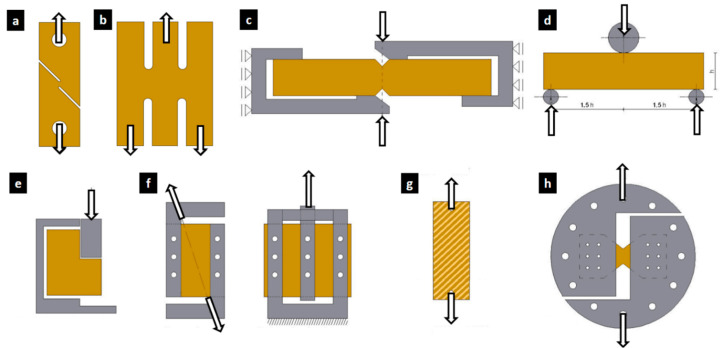
Specimen geometries for shear testing under different loads (arrows): (**a**) ASTM B831 standard [39,47], (**b**) Miyauchi [46], (**c**) Iosipescu [48], (**d**) short flexural beam, (**e**) block type (ASTM D143 [49]; ASTM D905 [50]; ASTM D1037 [35]), (**f**) two- and three-rail device [35], (**g**) ASTM D3518 (45° off-axis tensile) [51,52], and (**h**) Arcan fixture [53,54,55].

The samples commonly used to characterize wood and wood-based materials in a shear stress state are shown in Figure 1. For the design of the shear samples, the geometry and method of the Miyauchi shear test (Figure 1b [46]) are used as a reference. The specimen displays two shear zones from tensile loading in opposite directions between the inner and outer bars, thus generating shear stress between the connections of the three parallel bars. This sample is modified to avoid using special fixtures, facilitate the installation of the sample in the testing device, and apply compressive loads while maintaining a shear stress state (τ) between the bars. Several design iterations from the initial proposed specimen with dimensions of 900 mm height and 440 mm width were reduced to 200 mm height and 91 mm width. Several proposals were discarded for characterizing the 3 orthogonal shear directions, shear modulus, and shear strength.

**Figure 2:**
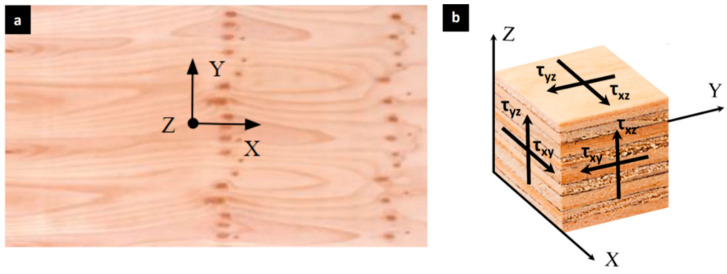
(**a**) Orthogonal directions in plywood panel. (**b**) Shear components of the stress tensor in Cartesian coordinates on a representative thickness element of the plywood panel.

Figure 2 shows the shear components (τ) of a stress tensor in orthogonal coordinates of a plywood panel. The X direction is oriented to the grain of the panel, the Y direction is perpendicular, and the Z direction is through thickness (out-of-plane) shear.

All the specimen geometries ensured the onset of failure within the gauge zone directly related to the shear stress. Additionally, specimens with knots in the failure zone or manufacturing imperfections were excluded to avoid non-presentative behavior.

The fabrication of all miniaturized specimens for orthotropic shear characterization involved executing different manufacturing toolpaths using a CNC router. A representative extraction from the boards combined and distributed the specimen locations within the samples as indicated in the EN 789 standard [37]. Finally, the next sections provide a summary of the new geometries and corresponding manufacturing toolpaths via the CNC router for all miniaturized specimen models used in the orthotropic shear characterization tests.

### 2.2. Radiata Pine Plywood Samples

Two commercially available plywood boards in the Chilean national market were used to manufacture the shear samples. The plywood used has a nominal thickness of 18 mm and is composed of 7 layers of rotary-cut *Pinus radiata* veneers arranged perpendicular to each other. The density is approximately 500 kg/m^3^. The two board samples are named hereafter F1 and F2 and are used as as-received raw material for shear sample manufacturing. From the initial characterization using datasheets and visual inspections, the main identified difference was the adhesive type. The F1 sample was based on phenol–formaldehyde adhesive, while F2 used an informed bioadhesive. The surface dimensions of the plywood boards were 1220 mm × 2440 mm. Both commercial panels complied with the PS1-22 standard [26].

**Figure 3:**
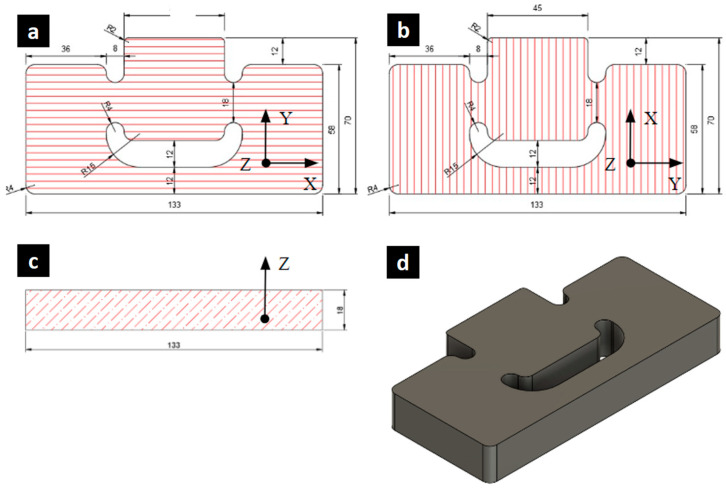
Proposed geometry for the in-plane shear test samples: (**a**) front view of τ_xy_, (**b**) front view of τ_yx_, (**c**) top view, and (**d**) isometric view of the samples.

Figure 3 shows the geometry of the shear test samples in the τ_xy_ and τ_yx_ directions. The samples, modified from the Miyauchi shear test (Figure 1b), had dimensions of 133 mm × 70 mm in length, with a double test zone of 18 mm × 18 mm. The shear samples featured increased cross-sectional areas in their three pillars to avoid combined stresses in the shear zone.

**Figure 4:**
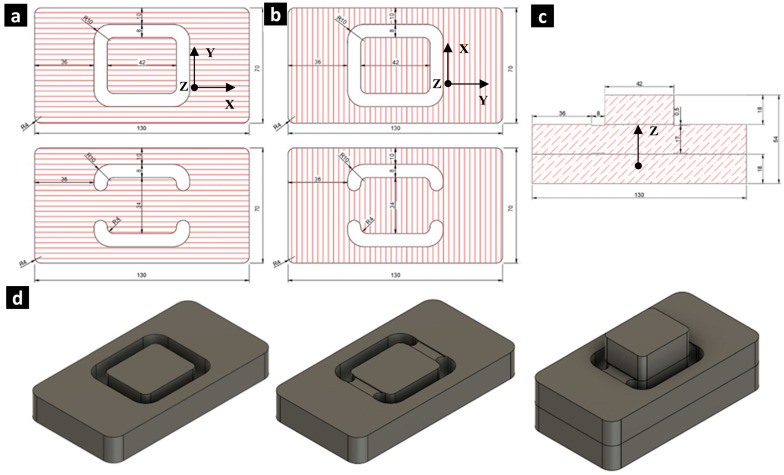
Proposed geometry for through-thickness shear test samples: (**a**) top view for τ_xz_, (**b**) top view for τ_yz_, (**c**) front view, and (**d**) isometric view of the assembly (right), including the outer lower piece and inner upper piece (left) and the piece with a notch in the test zone (middle).

Figure 4a,b details the geometry of the shear test samples for the shear stress directions τ_xz_ and τ_yz_, respectively. The specimen geometry consisted of three elements bonded with wood adhesive to prevent slippage or human error during the handling and installation of the samples in the testing machine. The test area included a uniform 8 mm wide and 0.5 mm deep notch to facilitate shear failure in the studied zone. The test area mirrors the geometry of the τ_xy_ and τ_yx_ shear samples, where, in this case, a normal and uniform force is applied to the top face of the section with dimensions of 42 mm × 34 mm or the upper block detailed in Figure 4c,d.

### 2.3. Shear Strength Characterization Tests

The mechanical tests were conducted in a temperature-controlled environment (20 ± 2 °C), and the average moisture content of the samples was 10.50% ± 0.50%. Six shear samples were tested to evaluate the stress states τ_xy_, τ_yx_, τ_xz_, and τ_yz_ at a constant strain rate of 0.001 s^−1^ on an INSTRON 3369 universal testing machine with a maximum capacity of 50 kN.

The shear modulus (Gs) was determined using Equation (1), where τ_10%Fmax_ and τ_40%Fmax_ correspond to 10% and 40% of the maximum stress (τ_Fmax_), respectively, and the values of ɛ_10%_ and ɛ_40%_ are associated with the values of τ_10%_ and τ_40%_, respectively. Equation (2) shows that the estimation of the shear strain (ɛ) is associated with the deformation angle ɣ, representing the relationship between the displacement detected by the testing machine and the 8 mm distance separating the compressed section and the supporting section connected through the shear zone:

(1) $$G_{s}=\frac{{\Delta \tau }_{s}}{2\Delta \varepsilon }=\frac{\left(\tau_{40\%\mathrm{Fmax}}-\tau_{10\%\mathrm{Fmax}}\right)}{2\left(\Delta_{40\%\mathrm{Fmax}}-\Delta_{10\%\mathrm{Fmax}}\right)}$$

where $\Delta \tau_{s}$ corresponds to the stress increment between 10% and 40% of τ_Fmax,_ and where Δε refers to the strain variation observed at 10% and 40% of τ_Fmax_.

(2) $$ɣ=\frac{d}{l}$$

d corresponds to the displacement measured by the testing machine (mm), and l is the perpendicular distance between sections connected by the shear failure zone (mm).

**Figure 5:**
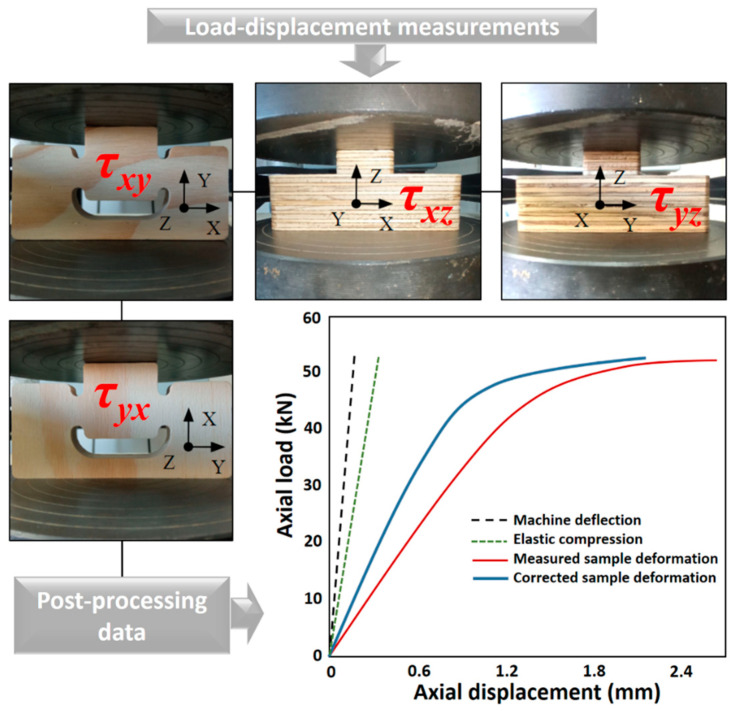
Mounting of the manufactured shear samples on a universal testing machine with compression plates in the τ_xy_, τ_yx_, τ_xz_, and τ_yz_ directions.

A curve correction procedure was implemented following the methodology detailed in Tuninetti et al. [56], which allowed for displacement adjustments by accounting for the combined deformations of the uniaxial machine devices and the specimen, as previously applied by Tuninetti et al. [57]. Figure 5 illustrates the procedure used to correct the data, showing the force-displacement curve obtained during the compression test of a sample subjected to loading in the corresponding deformation direction. The force-displacement curves obtained from plate-plate compression or machine flexibility tests and the corrected force–displacement curves are also presented. This approach improves the accuracy of the results used to determine plywood panels’ local and average properties.

### 2.4. Manufacturing of Miniaturized Shear Test Samples in Three Plywood Directions

**Figure 6:**
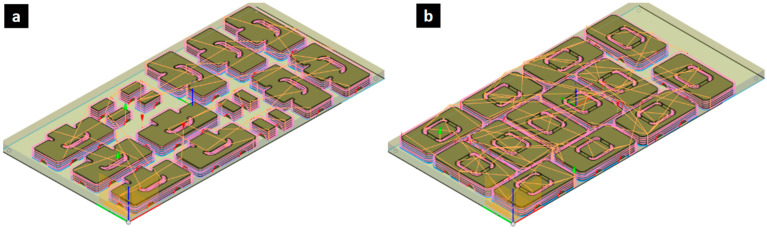
Toolpaths used for the fabrication of shear samples via a CNC router: (**a**) aamples for shear testing in xy–yx direction, and (**b**) samples for xz–yz shear direction.

The toolpaths for manufacturing via a CNC router, along with the positioning and orientation of the samples on the plywood boards, were carefully considered to optimize material usage and minimize waste during the manufacturing process. Figure 6a shows the toolpath for the samples characterizing S_xy_ and S_yx_ shear strength, arranged to efficiently utilize the available board space. Figure 6b shows the toolpath used for the S_xz_ and S_yz_ testing, which involves the optimization of the tool movements and sequences to achieve the fastest possible fabrication times while maintaining the required geometric accuracy. A ¼′′ diameter flat-end mill for wood applications was used with the following manufacturing parameters: spindle speed of 5000 rpm, cutting feed rate of 2400 mm/min, ramp feed rate of 800 mm/min, and a maximum cutting depth of 4 mm. These toolpath optimizations were crucial steps to ensure the accurate and repeatable manufacture of the miniaturized shear test samples across the various orthotropic directions of the plywood panels.

**Figure 7:**
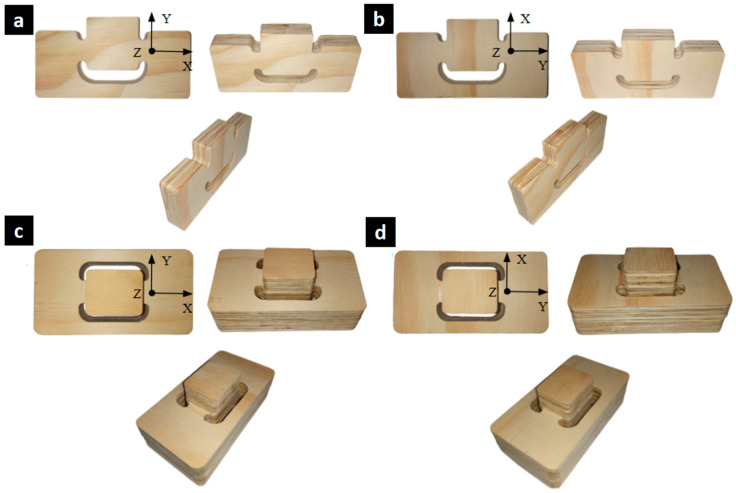
Type of samples prepared for shear tests in the direction of stress–strain: (**a**) τ_xy_, (**b**) τ_yx_, (**c**) τ_xz_, and (**d**) τ_yz_.

Figure 7c,d shows the fabricated samples for shear tests in the τ_xz_ and τ_yz_ directions. During the manufacturing process, a reduction in the cross-sectional area, proposed to achieve a higher stress concentration in the study zone, proved to be more challenging than anticipated. This is because the thickness tolerances of the boards exceeded the proposed notch depth by more than 10 times. Additionally, the outer veneers experienced the greatest thickness reduction due to grinding, primarily affecting the veneer on the board’s surface. As a result, the proposed notch primarily removed the outer veneers, excluding specimens with a significant reduction in cross-sectional area.

## 3. Results and Analysis

**Figure 8:**
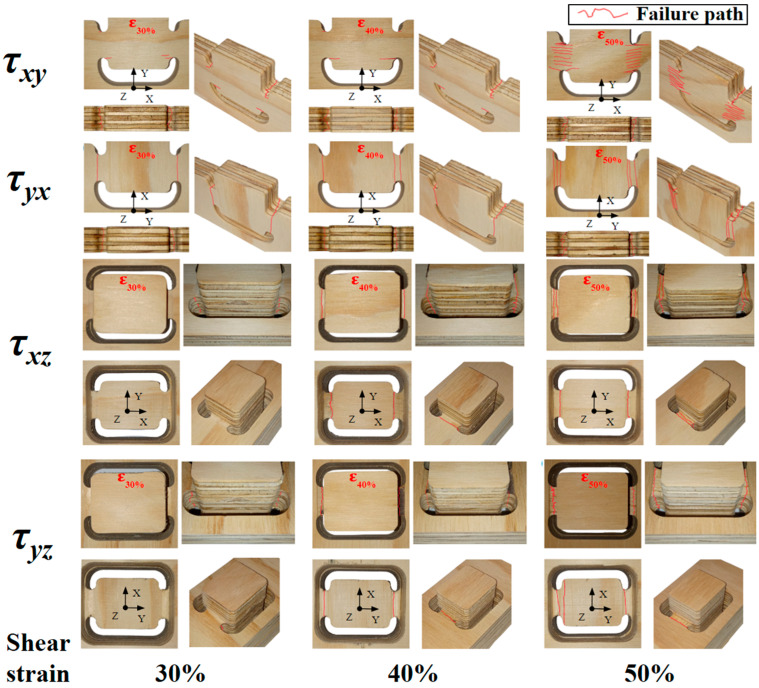
Deformation evolution at 30%, 40%, and 50% shear strain reflects the failure modes in the three tested orthotropic directions (τ_xy_, τ_xz_, and τ_yz_) for 18 mm thick plywood panels. The shear test in the τ_yx_ plane is also included for comparison with τ_xy_.

**Figure 9:**
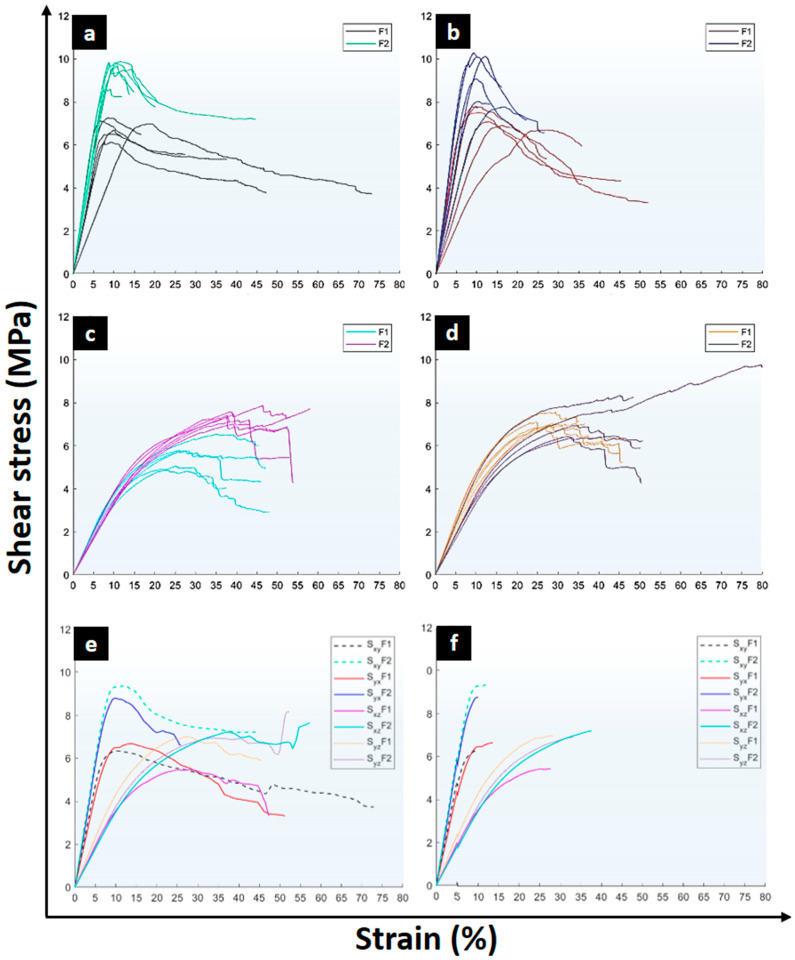
Shear stress–strain curves of plywood of investigated samples F1 and F2: (**a**) τ_xy_, (**b**) τ_yx_, (**c**) τ_xz_ and, (**d**) τ_yz_, (**e**) average curves of the damage zone and, (**f**) average curves until the maximum shear stress.

Figure 8 shows the progression of the deformed samples at 30%, 40%, and 50% shear-strain. The characteristic failure modes under shear in the three orthogonal planes τ_xy_, τ_xz_, and τ_yz_. τ_yx_ was included to compare τ_xy_ with τ_xy_ samples_._ These failures are correlated with the corresponding stress‒strain curves obtained from the shear tests (Figure 9).

### 3.1. Failure Mechanisms in Orthogonal Shear Planes

Figure 9a shows the stress‒strain curves obtained from the shear tests in the τ_xy_ direction. These curves reveal a homogeneous maximum strength, although clear differences between manufacturers are found. F2 samples achieved higher strength. The elastic region associated with the shear modulus shows greater variability in sample F1, with one specimen, in particular, showing a low slope, resulting in a significant reduction in the shear modulus, with a value of 0.049 GPa, compared with the second lowest recorded value of 0.087 GPa. After reaching the ultimate strength, samples experience an abrupt decrease in load-bearing capacity followed by a stabilization once the stress is reduced by 20% to 25%. Figure 8 supports this analysis since 30% of deformation failures observed on the outer faces of the samples are minor. As the deformation increases, fibers perpendicular to the applied force determine the ɣ associated with the deformation and the shear modulus. On the other hand, the veneers with fibers aligned with the direction of the force exhibit fiber separation fractures (top view).

Figure 9b shows the stress–strain curves obtained from the shear tests in the τ_yx_ direction. These curves should provide similar behavior to τ_xy_ for ideal shear conditions. Note that after failure, cracks appear and grow out of the shear gage zone (reduced section); this cannot be considered shear damage. This could be seen as a limitation of the application of this sample for shear damage characterization; however, as only inverse modeling provides accurate data, this sample shows the potential for this application. F2 samples exhibit higher maximum strengths in this direction. However, the elastic region shows greater variability. A difference is observed between the lowest and highest shear moduli: 0.044 and 0.073 GPa compared to 0.060 and 0.099 GPa for F1 and F2, respectively. In the non-linear phase, the pronounced drop in shear stress shows similar trends for both sample groups F1 and F2, although without the stress stabilization observed in the τ_xy_ direction. This latter difference is clearly attributed to the crack growth and orientations of the veneers and wood grains. As crack growth followed a path toward a zone where no deformation occurs (far from the gauge zone) for the τ_yx_ samples (Figure 8), the damage stress‒strain curve stabilized.

Homogeneous elastic stress–strain relationships are found for the τ_xz_ direction (Figure 9c. However, the development of strength until the maximum value follows a variable behavior, particularly in F1 samples, with average strength lower than F2. Non-linear strains develop early, as reflected in the reduction in the slope of the stress‒strain curves after the elastic region up to the maximum stress. This phenomenon can be explained by analyzing the composition of the veneers in the plywood panels. In this test orientation (Figure 8, τ_xz_, at 30%, 40%, and 50%), only four of the seven veneers composing the panel resist in a direction normal to the cross-section subjected to shear. Figure 8 shows that at τ_xz_, the three intermediate veneers, which are parallel to the cross-section subjected to shear stress, exhibit fractures from the beginning of the test, whereas the four perpendicular veneers show visible failures starting at 40% deformation.

Figure 9d presents the stress–strain curves of the samples subjected to shear in the τ_yz_ direction. The results indicate that both F1 and F2 samples exhibit linear behavior and maximum strength similarities. The similarity in the behavior of the samples from different manufacturers can be attributed to the fact that, in this case, the three inner veneers are perpendicular to the cross-section subjected to shear stress. Figure 8 shows that, from early deformations, the four veneers parallel to the cross-section subjected to shear experience failure, whereas the other three veneers only show visible failures.

Preliminary results from another research group show that the strain distribution from finite element analysis in similar sample geometries is not homogeneous [39]. This behavior is expected for similar specimens’ single shear or plane strain testing. Previous work includes a correction factor as a function of material properties to slightly increase accuracy. Further studies could focus on investigating these corrections and edge effects that could affect the accurate shear response and evaluate the quality of the deformations with appropriate orthotropic models or by image correlation. Note that even in the case of tensile, correcting for localized thinning and inhomogeneous deformations or compression of cylindrical specimens where friction affects the expected stress uniaxiality is necessary. These edge effects cannot be avoided, and they clearly generate a negligible associated error compared to the dispersion of the plywood response due to its manufacturing nature and components. A solution to be investigated in the future is to determine the experimental shear response using inverse finite element modeling, which leads to a more complex analysis with more expensive computational times that could not be justified if previous work has identified small errors due to edge effects and quasi-homogeneous stress and strain fields. However, improving the accuracy of the results could be interesting for science and knowledge generation.

### 3.2. Representative Shear Response in Plywood Panels

Figure 9e shows the average shear plane stress–strain curves for τ_xy_, τ_yx_, τ_xz_, and τ_yz_, whereas Figure 9f provides the average curves until the maximum shear stress is reached to reduce the interference associated with the overlap in the non-linear ranges following the maximum stress. For the τ_xy_ and τ_yx_ samples, the maximum stress condition is reached at deformations close to 10%, with F2 exhibiting the highest strength. From the summary of the results given in Table 1, high variability in specimen performance can be identified, with trends classified into two categories. The first trend corresponds to the τ_xy_ and τ_yx_ directions, which show the development of maximum stress under primarily elastic conditions with high stiffness. The second category includes τ_xz_ and τ_yz_, which exhibit a reduced rate of stress change with strain until reaching their maximum stress, indicating that these samples achieve their maximum strength under high-non-linear conditions. Figure 9f, the representative stress–strain curves for the τ_xy_, τ_yx_, τ_xz_, and τ_yz_ orientations up to the maximum strength are also given to highlight the differences in the deformation responses obtained depending on the test direction and manufacturer. τ_xy_ shows average deformations of 10.1% and 10.5% at the point of maximum stress for F1 and F2, respectively. In the τ_yx_ direction, the average deformation increases to 11.4% and 14.5% for each manufacturer. In the τ_xz_ direction, the deformations at the maximum stress increase significantly, reaching values of 27.0% and 46.3%, which also indicates differentiation between the origin of the sample. This trend continues, with average deformations of 28.8% and 47.1% for F1 and F2, respectively. However, it is important to note that the results from F2 in this latter orientation may show more variability. Nevertheless, in all the cases, F1 samples present lower deformation values at maximum strength.

The variability between the average maximum stresses provided by the representative curves of F1 and F1 yields the shear properties of the panels in Table 1, showing mean and standard deviation values for each shear direction. Significant differences between F1 and F2 are assessed using t-tests and Mann–Whitney U test. F1 samples suggest homogeneity in three of its four directions: τ_xy_, τ_yx_, and τ_yz_, whereas F2 samples indicate paired behavior (Table 1) between the trends in τ_xy_ and τ_yx_ and τ_xz_ with τ_yz_. Significant differences derived from statistical analysis between F1 and F2 are determined for ultimate shear strength in τ_xy_, τ_yx_, and τ_xz_. At the same time, significant differences in shear modulus are found for τ_yz_.

The differences between the stiffness and maximum strength of the τ_xy_ and τ_yx_ samples compared with those of the τ_xz_ and τ_yz_ samples can be attributed primarily to the plane of the veneers and the interaction with the adhesive. As veneers are generally stiffer and more brittle than adhesives, the adhesive layers flow more easily, which explains the more elastic and larger non-linear behavior with lower strength in the z-direction (through-thickness) than in the plane shear direction (x and y). The primary identified difference between the composition of F1 and F2 samples, which can justify the main stress–strain behavior observed, are the informed bonding nature. Phenol‒formaldehyde adhesive for F1 and bioadhesive for F2. This was finally confirmed with a shear modulus of τ_yz,_ showing a significant difference between F1 and F2 samples. However, even if nanomechanical testing could provide additional data to exclusively identify the properties of the adhesives and veneers separately, a composite model and orthogonal failure criteria with crack prediction modeling would be required to properly identified the specific nature of the stress–strain differences and crack patterns. Additionally, clear information from manufacturing methods and controlled variables, including the history of wood logs and three origins, could be required to include these variables that can affect the plywood properties. However, the goal of the study is to accurately characterize the shear loading behavior of plywood in the three directions, including the variability of the response, to include this information in plywood prediction models properly.

## 4. Method Limitations and Further Research

This section discusses the limitations of the current study and provides an orientation for future research focusing on improving shear properties determination using miniaturized samples to apply this data to engineering practices.

### 4.1. Numerical Modeling for Enhanced Accuracy of Shear Property Determination

The proposed sample geometry and existing literature generally lack local strain deformation values for determining the accuracy of identified properties. For material models of plywood and digital modeling using finite element simulations, the local properties are essential to compute the global properties in plywood applications. Previous studies on similar sample shapes demonstrated that edge effects of sample geometry influence the required homogeneous strain for accurate shear stress–strain behavior. The use of digital image correlation could confirm this effect; however, finite element modeling is required to quantify the associated errors from non-inhomogeneity of strain. For instance, the applied experimental method could be verified in the digital modeling of the experimental sample by comparing the computed values from the analytical expression with the numerical values from the model. In addition, to enhance the accuracy in the identification of parameters, inverse modeling of homogeneity could be adjusted to find the exact property, with consideration of edge effects. In addition, to quantify the accuracy, improve existing methods, or demonstrate superiority or suitability for specific property calibration, comparison informed from finite elements simulations can provide the required information to select the most adequate method.

### 4.2. Upscaling the Miniaturized Shear Test Approach for Plywood

Upscaling the miniaturized shear test approach for plywood presents both opportunities and challenges due to the inherent heterogeneity of the material. While the miniaturized method facilitates increased sample sizes and potentially more efficient material characterization, the layered structure and variability of wood properties in plywood necessitate careful consideration. As the sample size increases, the influence of local variations in wood characteristics and adhesive lines may diminish, leading to a more representative measure of overall shear behavior. However, the appropriate representative volume element (RVE) is essential; a larger RVE may be required for larger samples to capture the full range of material variability, which could impact the observed shear properties. Additionally, edge effects, which could be significant in smaller samples, may influence large-scale results differently. Correlating findings from scaled-up miniaturized tests with conventional full-scale tests is essential to validate the method’s applicability and establish its limitations in accurately predicting plywood shear behavior at larger scales. Further research should explore the interactions between sample size, RVE, and edge effects to refine the scaled-up method, ensuring its reliability for assessing the structural performance of plywood in real-world applications.

### 4.3. Applying the Method for Anisotropy Characterization of Wood and Wood-Based Products

Analysis of the anisotropic response of plywood, which results from its layered structure and the different properties of wood in the longitudinal and transverse directions, can be applicable to other wood-based composites. This study’s miniaturized shear testing methodology could be adapted for materials such as cross-laminated timber or laminated veneer lumber, which have similar structural anisotropic behavior. For example, research on CLT often focuses on characterizing its rolling shear properties, which are critical for structural design [58]. Address rolling shear and comparing the miniaturized testing methodology could potentially provide additional data for validation. Similarly, studies of LVL often investigate the influence of veneer orientation and adhesive properties on its mechanical performance [59]. Comparing the results of this study with those of LVL could also provide additional data for model calibration and behavior of engineered wood products.

Furthermore, exploring the relationship between the observed anisotropy and the moisture content of the material, as investigated in some studies on plywood and other wood-based composites [58,60], could provide a more comprehensive understanding of the material’s behavior under different environmental conditions. Finally, linking the results to broader research on wood biomechanics and the influence of microstructural features on macroscopic properties could further enrich the analysis and extend its applicability to a wider range of wood materials [61,62].

### 4.4. Moisture and Temperature Impacts on Plywood: An Identified Research Gap

The influence of moisture content and temperature on plywood properties is well-accepted in research and engineering. ASTM D4442 [63] provides methods for measuring moisture content, while BS EN 321 [64] provides guidelines for evaluating the effects of moisture and temperature cycling on the strength of wood-based panels. However, research quantifying the specific individual or combined effects of moisture and temperature on shear strength is limited. Karshenas and Feely [65] investigated the structural properties of used plywood with a moisture content of 17%. Although their study does not specifically analyze the effects of moisture and temperature, it recognizes their significant influence on plywood performance over time and emphasizes the importance of considering the historical exposure of plywood to environmental conditions. Gerhards [66] studied the properties of solid wood, concluding that increased moisture content generally reduces strength. Higher temperatures can exacerbate this effect, especially above the fiber saturation point. Conversely, lower moisture levels and temperatures generally increase strength, although excessively low moisture can make wood brittle. Although focused on solid wood, these principles also apply to the plywood veneers. Stadlmann et al. [67] studied the effects of natural and accelerated weathering on solid wood and found that both resulted in a reduction in mechanical properties due to changes in wood structure and salt diffusion. These results suggest that plywood veneers may also degrade under similar moisture, temperature, and exposure conditions, emphasizing the need to consider these environmental factors when evaluating the durability of plywood. Wind [68] investigated the bonding performance of mass plywood (MPP) panels under varying moisture and temperature conditions and found that the melamine formaldehyde adhesive performed consistently within an approximate range of 10–16% moisture content and 60–90 °F. Failures occurred predominantly in the wood substrate rather than the adhesive.

While there is some data on the effects of moisture and temperature on solid wood from Gerhards [66] and Stadlmann et al. [67], specific combined effects on plywood shear strength remain largely unexplored. In addition, Karshenas and Feely [65] focus on using plywood in uncontrolled environments, and Wind [68] emphasizes bond performance rather than overall strength. Therefore, further research is needed to better understand how moisture content and temperature affect plywood properties, including extreme environments of 80 °C for specific applications like roof sheathing [69].

To investigate the effects of moisture and temperature on plywood properties, experiments could test samples over 8–18% moisture and −20 to 80 °C, accounting for varying humidity, rain, and temperature. Relevant standards and proposed methods could assist in measuring the mechanical properties. Factors like wood species, adhesives, and construction could also be analyzed. This understanding has practical applications across various industries. In engineering design, this knowledge can inform the selection of appropriate plywood grades, adhesives, and protective measures based on expected environmental conditions and accurate simulation tools. In construction, it is crucial to ensure proper storage and handling to prevent moisture absorption before installation and consider ventilation and moisture barriers in building design. Furthermore, this understanding can be applied to any application involving plywood exposed to varying moisture and temperature, such as furniture manufacturing, packaging, and transportation.

## 5. Conclusions

The present study provides a comprehensive experimental investigation of the shear properties of plywood across its three main orthotropic directions: τ_xy_, τ_xz_, and τ_yz._ Shear tests were performed on miniaturized samples, revealing key findings about the plywood behavior:

- The τ_xy_ and τ_yx_ samples, representing the parallel in-plane shear directions, exhibited a more elastic and ductile response. These samples displayed the highest mean ultimate shear strengths, at 6.8 MPa and 7.3 MPa, respectively, as well as the highest shear moduli of 0.095 GPa and 0.087 GPa.
- In contrast, the τ_xz_ and τ_yz_ samples, representing the cross-plane shear directions, demonstrated a more plastic deformation behavior. These samples reached their maximum strengths (5.6 MPa and 7.1 MPa, respectively) at significantly higher deformations (27.0–47.1%) due to the complex interaction between the wood veneers and adhesive layers.
- The plywood panels with bioadhesive exhibited greater overall strength in the parallel in-plane directions (τ_xy_ and τ_yx_) but also showed higher variability in the shear modulus compared to the other orientations.
- These findings highlight plywood’s orthotropic and heterogeneous nature, particularly at smaller scales. The use of miniaturized samples enabled increased sample sizes, providing valuable data for developing and validating finite element models of plywood behavior.

To advance the understanding of plywood behavior, future research should focus on evaluating the identified orthotropic characteristics and data variability at larger scales through finite element modeling. Additionally, the applicability of the miniaturized sample approach to other engineered wood products and the comparison with standardized testing protocols should be explored by experimental or numerical analysis for validation. The quantitative influence of moisture content and temperature on plywood behavior and anisotropic properties should also be explored. Integrating these factors into finite element models, supported by digital image correlation data on local strain distributions, can substantially enhance the predictive capabilities of simulations under varying environmental conditions.

## Figures and Tables

**Table 1:** Summary of shear test results, mean and standard deviation (SD), including normality and significant differences between sample groups and directions.

Shear Direction	Property	Statistical Values	Number of Samples	Samples Group		Normality	Significant Difference
				F1	F2		
τ_xy_	τ_Fmax_ (MPa)	Mean	6	6.788	9.561	Only for F1	Yes
		SD		0.423	0.488		
	G_s_ (GPa)	Mean		0.095	0.120	Yes	No
		SD		0.029	0.011		
τ_yx_	τ_Fmax_ (MPa)	Mean		7.291	9.231		Yes
		SD		0.463	1.121		
	G_s_ (GPa)	Mean		0.087	0.113		No
		SD		0.033	0.028		
τ_xz_	τ_Fmax_ (MPa)	Mean		5.636	7.393	Only for F1	Yes
		SD		0.587	0.279		
	G_s_ (GPa)	Mean		0.039	0.036	Yes	No
		SD		0.003	0.003		
τ_yz_	τ_Fmax_ (MPa)	Mean	6 (F1) 5 (F2)	7.074	7.591	Only for F2	
		SD		0.261	1.447		
	G_s_ (GPa)	Mean		0.046	0.039	Only for F1	Yes
		SD		0.005	0.005		

## Data Availability

The original contributions presented in the study are included in the article, further inquiries can be directed to the corresponding author.
